# Draft Genome Sequence of *Janthinobacterium* sp. Strain SLB01, Isolated from the Diseased Sponge *Lubomirskia baicalensis*

**DOI:** 10.1128/MRA.01108-19

**Published:** 2019-11-07

**Authors:** Ivan S. Petrushin, Sergei I. Belikov, Lubov I. Chernogor

**Affiliations:** a Limnological Institute, Siberian Branch of the Russian Academy of Sciences, Irkutsk, Russia; b Irkutsk State University, Irkutsk, Russia; Georgia Institute of Technology

## Abstract

The draft genome sequence of Janthinobacterium sp. strain SLB01, a violacein-producing psychrotolerant bacterium isolated from the diseased sponge Lubomirskia baicalensis, was determined. We identified five genes encoding VioA, VioB, VioC, VioD, and VioE proteins related to violacein biosynthesis that were like those identified in published Janthinobacterium lividum strains MTR and RIT308.

## ANNOUNCEMENT

A significant increase in the number of opportunistic microorganisms, including members of the family Oxalobacteraceae, was detected by the analysis of 16S rRNA genes in diseased freshwater sponges during their mass death (1, 2). In this study, a violet-pigmented Janthinobacterium sp. strain was isolated on Luria-Bertani (LB) broth medium agar plates (diluted 1/10; temperature, 15°С) from the diseased sponge Lubomirskia baicalensis. The sponge was collected in February 2015 from Lake Baikal in the Olkhon Gate Strait, Russia (53°02′21″N, 106°57′37″ E), at a depth of 10 m (water temperature, 3 to 4°С). The genus *Janthinobacterium* includes rod-shaped, Gram-negative, aerobic bacteria that produce the purple pigment violacein (3, 4). Violacein has been known to have antimicrobial and antiviral effects (5). To date, genomes of many environmental isolates of *Janthinobacterium* spp. from ice, water, sediments, and soils have been sequenced. This study, for the first time, isolated *Janthinobacterium* sp. strain SLB01 from a diseased freshwater sponge.

Genomic DNA was isolated following a standard bacterial DNA isolation cetyltrimethylammonium bromide (CTAB) protocol (https://jgi.doe.gov/user-programs/pmo-overview/protocols-sample-preparation-information/jgi-bacterial-dna-isolation-ctab-protocol-2012/). The sequence library was prepared with the Nextera XT DNA library preparation kit (Illumina, USA). Genomes were sequenced on the Illumina MiSeq platform using v2 paired-end chemistry (2 × 250 bp, 12,099,942 reads total). The bacterial genus of the isolate was identified by analysis of sequencing data of the 16S rRNA gene fragments. The reads were error corrected with SPAdes and the built-in BayesHammer module (quality threshold, 98%), and a draft assembly was built using SPAdes version 3.11.0 with default settings (6). This draft assembly contained 298 contigs with an *N*_50_ value of 609,552 bp, a largest contig length of 1,308,902 bp, and genome coverage of 180×. The resulting contigs were ordered with Ragout version 2.2 with default settings (https://github.com/fenderglass/Ragout) (7), using the *Janthinobacterium* sp. strain LM6 chromosome (GenBank accession number CP019510) as the reference genome and 21 contigs from the draft assembly. The final assembly contained 2 scaffolds, with a total genome size of 6,467,981 bp and a GC content of 62.63%. Automated annotation for the genome was performed by the PROKKA pipeline (8) and the NCBI Prokaryotic Genome Annotation Pipeline (PGAP) (9). This assembly contained 5,591 genes encoding 5,463 protein-coding sequences, 76 tRNAs, 4 noncoding RNAs (ncRNAs), 5 rRNAs, and 43 pseudogenes, as identified by PGAP.

We identified five genes similar to violacein biosynthesis genes *vioA*, *vioB*, *vioC*, *vioD*, and *vioE*, which are present in the genomes of Janthinobacterium lividum strains MTR and RIT308 (10, 11), as well as the genes encoding α-hydroxy ketone quorum-sensing regulons such as CAI-1/LAI-1-like autoinducer synthase JqsA, CAI-1/LAI-1-like autoinducer sensor kinase/phosphatase JqsS, and the response regulator JqsR. In addition, we found genes associated with twitching motility (encoding tip-associated adhesin PilY1 and type IV pilus secretin PilQ PilW family protein) and the T6 secretion system (encoding membrane subunit TssM, baseplate subunits TssE, TssF, and TssK, and lipoprotein TssJ), which can be associated with the pathogenicity of *Janthinobacterium* sp. isolates for sponges. This knowledge is useful for understanding the role of *Janthinobacterium* spp. in the disease and death of Baikal sponges.

### Data availability.

This whole-genome shotgun project has been deposited at DDBJ/ENA/GenBank and in the Sequence Read Archive (SRA) under BioProject number PRJNA565495, BioSample number SAMN12748851, and accession number VZAB00000000. The versions described in this paper are the first versions.
